# Data on RNA-seq analysis of Drosophila melanogaster during ageing

**DOI:** 10.1016/j.dib.2021.107413

**Published:** 2021-09-22

**Authors:** Morteza Bajgiran, Azali Azlan, Shaharum Shamsuddin, Ghows Azzam, Mardani Abdul Halim

**Affiliations:** a School of Biological Sciences, Universiti Sains Malaysia, Gelugor, Penang 11800, Malaysia; b USM-RIKEN Interdisciplinary Collaboration for Advanced Sciences, Universiti Sains Malaysia, Gelugor, Penang 11800, Malaysia; c School of Health Science, Universiti Sains Malaysia Health Campus, Kubang Kerian, Kelantan 16150, Malaysia; d Biotechnology Research Institute, Universiti Malaysia Sabah, Jalan UMS, Kota Kinabalu, Sabah 88400, Malaysia

**Keywords:** RNA-seq, Transcriptome, Transcriptomics, Drosophila melanogaster, Ageing

## Abstract

Ageing is defined as gradual decline of physiological, cellular and molecular state of an organism with time. The age-associated cell dysfunctions usually cause chronic diseases such as diabetes, cancers and other age-related diseases. Many of the genes and pathways involved in ageing are conserved in different species. These genes and pathways have been categorised into nine cellular and molecular hallmarks, namely, genomic instability, telomere attrition, loss of proteostasis, mitochondrial dysfunction, epigenetic alterations, deregulated nutrient sensing, stem cell exhaustion, cellular senescence and altered intercellular communication. Despite countless studies on ageing, the molecular mechanism of ageing is poorly understood. Here, we performed genome wide transcriptome mapping of ageing process in *D. melanogaster.* In which, transcriptomic analysis conducted on the 1 day and 60 days flies. Illumina Hiseq platform were used to generate raw data. Afterwards, further analysis including differential expression analysis, GO classification and KEGG pathway enrichment analysis were performed. The raw data were uploaded to SRA database and the BioProject ID is PRJNA718442. These data provide the basis for future research in order to discover the genes and pathways involved in ageing.

## Specifications Table

**Table:** 

Subject	Ageing
Specific subject area	Transcriptomic changes during ageing, comparing expression changes that occur in ageing, investigating the involvement of molecular pathways in ageing
Type of data	RNA-seq data, figures, tables
How data were acquired	RNA sequencing by Illumina Hiseq platform Softwares: HISAT2, featurecount, edgeR, DAVID online tool
Data format	Raw (FASTQ), excel spreadsheet, image, table
Parameters for data collection	Total RNA extraction and sequencing of samples in two different conditions, namely, day 1 Drosophila melanogaster (young) and day 60 Drosophila melanogaster (old) performed.
Description of data collection	Total RNA was isolated using Trizol reagent and RNeasy MinElute Cleanup Kit. RNA quality was evaluated by electrophoresis, Nanodrop2000 and Agilent2100 Bioanalyzer. rRNA was removed and then samples prepared and sequenced.
Data source location	School of biological sciences, Universiti Sains Malaysia (USM), Malaysia (5.3557° N, 100.3012° E)
Data accessibility	Data can be accessed from NCBI SRA (BioProject ID: PRJNA718442) https://www.ncbi.nlm.nih.gov/bioproject/PRJNA718442.

## Value of the Data

• These data provide a comprehensive picture with a greater resolution of gene expression changes and the pathways involved in the process of ageing in *D. melanogaster*.
• The dataset and analysis provided here can be useful for researchers focusing on aging and age-related diseases such as Alzheimer, cancer, and cardiovascular diseases in *D. melanogaster*.
• Applying different workflows, the RNA-seq raw data provided here can be used for further analysis to investigate the role of coding and non-coding genes in ageing. Besides, the analysis provided here would shed light on potential genes and pathways involved in ageing process for further molecular research in order to find novel anti-ageing strategies and treatments for age-related diseases.

## 1 Data Description

To investigate changes in molecular landscape in ageing process, day 1 and day 60 flies of *D. melanogaster* were chosen as model system and RNA sequencing was done using Illumina Hiseq platform. Table 1 provides accession numbers and links for raw data generated by RNA sequencing. There are in total three paired end libraries for day 1, and three paired end libraries for day 60 flies. Raw reads generated was mapped by HISAT2 and differential expression analysis was performed using edgeR. Table 2 shows the summary of libraries statistics and mapping including number of raw reads, number of cleaned reads and mapping rates. Differentially expressed genes and their respective fold change and expression levels as count per million (CPM) are listed in supplementary 1. Differentially expressed genes, further, were chosen for GO classification and KEGG pathway analysis. The enriched GO terms featuring biological process, cellular component, and molecular functions and the number of differentially expressed genes related to those GO terms are presented in Tables 3–5, respectively. Table 6 shows the result of KEGG pathway enrichment analysis in day 60 compared to day 1 flies. Number of differentially expressed genes related to each KEGG pathway is provided in Table 6.

**Table 1:** Accession numbers and links for raw data of ageing D. melanogaster transcriptome at two time points; day 1 and day 60.

Sample	Accession number	Accession link
Day1 replicate 1	**SAMN18533764**	https://www.ncbi.nlm.nih.gov/biosample/18533764
Day1 replicate 2	**SAMN18533765**	https://www.ncbi.nlm.nih.gov/biosample/18533765
Day1 replicate 3	**SAMN18533766**	https://www.ncbi.nlm.nih.gov/biosample/18533766
Day60 replicate 1	**SAMN18533767**	https://www.ncbi.nlm.nih.gov/biosample/18533767
Day60 replicate 1	**SAMN18533768**	https://www.ncbi.nlm.nih.gov/biosample/18533768
Day60 replicate 3	**SAMN18533769**	https://www.ncbi.nlm.nih.gov/biosample/18533769

**Table 2:** Summary of libraries statistics and mapping results. Following the sequencing of 6 samples (three replicate for each time point), the reads were trimmed and mapped to OreR genome. All the libraries are paired-end and the length of reads is 151.

Library	%GC	Number of raw reads	Number of cleaned reads	Mapping rate
Day1 replicate 1	45	49210530	48883744	96.7
Day1 replicate 2	45	51138168	50773952	96.4
Day1 replicate 3	44	49970634	49629740	92.4
Day60 replicate 1	51	56678370	56334634	95.9
Day60 replicate 1	50	50948514	50564212	95.7
Day60 replicate 3	50	53317220	52898742	95.3

**Table 3:** Enriched GO terms featuring biological process. Significantly differentially expressed genes in day 60 compare to day1 are categorised into 27 GO terms featuring biological process with significant of *P*-value< 0.05. The number of differentially expressed genes related to the GO terms are presented as count with their respective *P*-value.

ID	GO term	Count	*P*-Value
GO:0022008	neurogenesis	502	1.97681854884323E-37
GO:0006357	regulation of transcription from RNA polymerase II promoter	149	3.59418611188958E-09
GO:0006367	transcription initiation from RNA polymerase II promoter	61	1.26263630284492E-07
GO:0006355	regulation of transcription, DNA-templated	311	2.8218671227443E-07
GO:0006351	transcription, DNA-templated	293	2.96938976281765E-07
GO:0046331	lateral inhibition	173	3.0790442508602E-07
GO:0000398	mRNA splicing, via spliceosome	184	3.25585534793413E-07
GO:0002181	cytoplasmic translation	89	3.68214156503802E-07
GO:0006909	phagocytosis	171	4.78720323420357E-07
GO:0009267	cellular response to starvation	84	1.5933196362708E-06
GO:0051301	cell division	63	4.11556745111304E-06
GO:0045944	positive regulation of transcription from RNA polymerase II promoter	182	4.63036305501604E-06
GO:0000381	regulation of alternative mRNA splicing, via spliceosome	66	5.11423501215597E-06
GO:0007095	mitotic G2 DNA damage checkpoint	65	6.95407725462918E-06
GO:0006366	transcription from RNA polymerase II promoter	61	7.73207377601407E-06
GO:0006281	DNA repair	78	8.80839391227024E-06
GO:0032543	mitochondrial translation	76	1.53778972185858E-05
GO:0006364	rRNA processing	43	1.56116871043411E-05
GO:0000122	negative regulation of transcription from RNA polymerase II promoter	122	2.48056411611E-05
GO:0008285	negative regulation of cell proliferation	41	3.15667448402814E-05
GO:0007067	mitotic nuclear division	114	4.17734461813615E-05
GO:0051297	centrosome organization	51	5.58585113504924E-05
GO:0006606	protein import into nucleus	30	6.02653445152604E-05
GO:0000462	maturation of SSU-rRNA from tricistronic rRNA transcript (SSU-rRNA, 5.8S rRNA, LSU-rRNA)	30	6.02653445152604E-05
GO:0051726	regulation of cell cycle	77	6.43923474482933E-05
GO:0008360	regulation of cell shape	88	8.33910875356639E-05
GO:0019233	sensory perception of pain	440	8.88303802438348E-05

**Table 4:** Enriched GO terms featuring cellular component. Significantly differentially expressed genes in day 60 versus day1 are categorised into 25 GO terms featuring cellular component with significant of *P*-value< 0.05. The number of differentially expressed genes related to the GO terms are presented as count with their respective *P*-value.

**ID**	GO term	Count	*P*-Value
GO:0005634	nucleus	1447	3.27917457291437E-20
GO:0071011	precatalytic spliceosome	141	2.51427093226526E-12
GO:0005737	cytoplasm	1203	1.23883014505389E-11
GO:0005875	microtubule associated complex	260	3.97418659471114E-11
GO:0071013	catalytic step 2 spliceosome	122	1.36639696939448E-10
GO:0005730	nucleolus	150	6.93025838400435E-08
GO:0012505	endomembrane system	177	2.8756207140686E-07
GO:0005622	intracellular	245	4.95714684182731E-05
GO:0005813	centrosome	84	7.7186956064813E-05
GO:0005739	mitochondrion	371	1.99536454552943E-04
GO:0005681	spliceosomal complex	45	3.58332448204499E-04
GO:0030532	small nuclear ribonucleoprotein complex	34	5.51522366369312E-04
GO:0000775	chromosome, centromeric region	37	9.26713989307439E-04
GO:0032040	small-subunit processome	32	9.98098412354131E-04
GO:0005819	spindle	45	0.001109693298146730
GO:0005635	nuclear envelope	45	0.001109693298146730
GO:0022625	cytosolic large ribosomal subunit	52	0.0014094411399342900
GO:0005840	ribosome	83	0.0017779517106270500
GO:0005654	nucleoplasm	123	0.0023704122216551300
GO:0043234	protein complex	61	0.004578248094672790
GO:0000922	spindle pole	31	0.004752710283904030
GO:0016020	membrane	296	0.006993145644730750
GO:0005747	mitochondrial respiratory chain complex I	41	0.0073329881920681800
GO:0005643	nuclear pore	33	0.008068390351287990
GO:0005912	adherens junction	44	0.008423004737610120

**Table 5:** Enriched GO terms featuring molecular function. Significantly differentially expressed genes in day 60 compare to day1 are categorised into 42 GO terms featuring molecular function with significant of *P*-value< 0.05. The number of differentially expressed genes related to the GO terms are presented as count with their respective *P*-value.

ID	GO term	Count	*P*-Value
GO:0005524	ATP binding	612	1.05918346247829E-12
GO:0005515	protein binding	527	5.96469126229741E-11
GO:0003676	nucleic acid binding	359	1.02969701996623E-09
GO:0008270	zinc ion binding	519	2.03153421977325E-09
GO:0003723	RNA binding	230	4.35245434691068E-08
GO:0005509	calcium ion binding	181	1.49307405316839E-07
GO:0046872	metal ion binding	519	2.59437961279962E-07
GO:0003713	transcription coactivator activity	54	3.1383537146616E-05
GO:0004386	helicase activity	47	5.61299888650304E-05
GO:0003682	chromatin binding	111	6.63049808227043E-05
GO:0000166	nucleotide binding	175	6.76405677489166E-05
GO:0003729	mRNA binding	143	1.03224400384053E-04
GO:0008017	microtubule binding	91	4.19831996266617E-04
GO:0004722	protein serine/threonine phosphatase activity	42	8.43623758676245E-04
GO:0004004	ATP-dependent RNA helicase activity	45	0.0011679797602636700
GO:0003954	NADH dehydrogenase activity	31	0.0013941636413593100
GO:0044822	poly(A) RNA binding	63	0.0015759251370228100
GO:0016887	ATPase activity	123	0.001611451461034100
GO:0001104	RNA polymerase II transcription cofactor activity	32	0.003781513889304200
GO:0003714	transcription corepressor activity	27	0.004411964739511650
GO:0004842	ubiquitin-protein transferase activity	143	0.004674282350421040
GO:0003684	damaged DNA binding	26	0.0058537637092831700
GO:0019843	rRNA binding	26	0.0058537637092831700
GO:0008134	transcription factor binding	72	0.012680359459747200
GO:0003743	translation initiation factor activity	48	0.01586414858335640
GO:0003924	GTPase activity	106	0.017835528186287700
GO:0003899	DNA-directed RNA polymerase activity	26	0.01786875512397270
GO:0051539	4 iron, 4 sulfur cluster binding	26	0.01786875512397270
GO:0003755	peptidyl-prolyl cis-trans isomerase activity	29	0.02168192134654930
GO:0016853	isomerase activity	29	0.02168192134654930
GO:0003705	transcription factor activity, RNA polymerase II distal enhancer sequence-specific binding	46	0.023058171236249100
GO:0051082	unfolded protein binding	45	0.02767670647492650
GO:0042393	histone binding	24	0.029256156587200600
GO:0016740	transferase activity	31	0.030883614952758100
GO:0003824	catalytic activity	98	0.03357953732093580
GO:0005484	SNAP receptor activity	23	0.037228197776595800
GO:0004693	cyclin-dependent protein serine/threonine kinase activity	15	0.038453595676428300
GO:0030515	snoRNA binding	15	0.038453595676428300
GO:0001075	transcription factor activity, RNA polymerase II core promoter sequence-specific binding involved in preinitiation complex assembly	19	0.03922287285579800
GO:0042803	protein homodimerization activity	110	0.043234103342660000
GO:0042623	ATPase activity, coupled	39	0.045560193420034400
GO:0000977	RNA polymerase II regulatory region sequence-specific DNA binding	48	0.04871352258031190

**Table 6:** KEGG pathway enrichment analysis. 10 KEGG pathways are significantly enriched by differentially expressed genes in day 60 compare to day 1 with significant of *P*-value< 0.05. The number of differentially expressed genes related to the pathway are presented as count with their respective *P*-value.

Term	Count	P-Value
Spliceosome	110	5.73666837052742E-04
DNA replication	34	0.0034766573188115000
Nucleotide excision repair	37	0.006074627455663300
Basal transcription factors	36	0.007531383638678190
Protein processing in endoplasmic reticulum	107	0.009490293966813040
mRNA surveillance pathway	61	0.022952383725918000
Mismatch repair	20	0.02518504502307330
Purine metabolism	110	0.02557908482786070
Fanconi anemia pathway	25	0.02684084015079530
Ubiquitin mediated proteolysis	82	0.0493859761510847

## 2 Experimental Design, Materials and Methods

### 2.1 Fly husbandry

Wild-type Oregon-R (OreR) (genotype: Oregon-R-P2; stock no.: 107294) from Kyoto Stock Center was used. The flies were maintained at 25 °C, 12 h light/dark cycle in a corn-based meal consists of 4% (w/v) corn starch, 5% (w/v) polenta, 10% (w/v) brown sugar, 0.7% (w/v) agar, 5% (w/v) yeast, 3% (w/v) nipagin and 0.7% (v/v) propionic acid.

### 2.2 Total RNA extraction, library construction, and RNA-seq

Equal number of male and female flies was used to extract the total RNA. A combination of Trizol reagent (Invitrogen, USA) and RNeasy MinElute Cleanup Kit (Qiagen, Germany) was used to extract the RNA. The flies were homogenized in 500 µL of Trizol reagent, then, a volume of 100 µL of chloroform was added into the mixture. The sample was thoroughly mixed and centrifuged at 10,000 xg for five minutes. A volume of 1000 µL of isopropanol was added into aqueous layer and thoroughly mixed. The sample was cleanup using MinElute Cleanup Kit according to manufacturer protocol. gDNA was removed using Turbo^TM^ DNase Kit (Thermo Fisher Scientific, USA). The quality of extracted RNA was assessed by agarose gel electrophoresis, Nanodrop2000 (Thermo Fisher Scientific, USA), and Agilent2100 Bioanalyzer (Agilent, USA). High quality RNA (≥ 5 µg; ≥ 200 ng/µL; OD260/280 = 1.8–2.2) will be used for library construction.

For library construction, standard Illumina protocol was employed. The first step involving the enrichment of mRNA using poly-T oligo attached magnetic beads. Then, the mRNA was fragmented using divalent cations. First strand cDNA synthesis was performed using SuperScript II followed by second strand. End repair was performed to remove any overhangs prior to adenylation of 3’ends. Then, adapter was ligated, and size selection (150–200 bp) was performed. The purified size-selected RNA was sequenced using Illumina Hiseq platform. Raw data generated was trimmed and cleaned by removing low quality reads and removing the adaptor.

### 2.3 Differential expression analysis

RNA-seq reads were aligned to the reference genome of *D. melanogaster by* using HISAT2 version 2.1.0 [1]. The genome was Drosophila_melanogaster.BDGP6.28.dna_sm.toplevel.fa.gz downloaded from Ensembl. Afterwards, in order to quantify the expression level of transcripts the alignment files generated by HISAT2 were used as inputs for featurecount [2]. These counts were then used as input for differential analysis using using edgeR [3]. The statistical program edgeR was analyzed in R/Bioconductor environment. FDR< 0.05 were set as the threshold for significantly differential expression genes [4].

### 2.4 GO classification and enrichment analysis

DAVID online tool was used to identify significantly enriched GO terms featuring biological process, cellular component, molecular function and KEGG pathways with corrected *P*-value less than 0.05 [5,6].

## Ethics Statements

All animal handlings complied with guidelines set forth by the National Institutes of Health for the care and use of laboratory animals, and the protocol of this study followed the National Institutes of Health guide for the care and use of laboratory animals (NIH Publications No. 8023, revised 1978) and Guide for the Care and Use of Laboratory Animals: Table 4 8th Edition.

## CRediT authorship contribution statement

**Morteza Bajgiran:** Methodology, Resources, Investigation, Formal analysis, Data curation, Writing – original draft, Writing – review & editing. **Azali Azlan:** Software, Data curation, Formal analysis. **Shaharum Shamsuddin:** Supervision, Funding acquisition. **Ghows Azzam:** Supervision, Funding acquisition. **Mardani Abdul Halim:** Conceptualization, Methodology, Resources, Investigation, Data curation, Writing – original draft, Supervision.

## Declaration of Competing Interest

The authors declare that they have no known competing financial interests or personal relationships which have or could be perceived to have influenced the work reported in this article.

## Appendix Supplementary materials

Image, application 1
